# Epidemiological, clinical, outcome and antibiotic susceptibility differences between PVL positive and PVL negative *Staphylococcus aureus* infections in Western Australia: a case control study

**DOI:** 10.1186/s12879-014-0742-6

**Published:** 2015-01-09

**Authors:** Peter Boan, Hui-Leen Tan, Julie Pearson, Geoffrey Coombs, Christopher H Heath, James Owen Robinson

**Affiliations:** Department of Microbiology and Infectious Diseases and PathWest Laboratory Medicine WA, Royal Perth Hospital, Wellington St, Perth, WA 6000 Australia; Australian Collaborating Centre for Enterococcus and Staphylococcus Species (ACCESS) Typing and Research, PathWest Laboratory Medicine WA and Curtin University of Technology, GPO Box X2213, Perth, WA 6847 Australia

**Keywords:** *Staphylococcus aureus*, MRSA, Panton Valentine Leucocidin, PVL

## Abstract

**Background:**

Panton Valentine Leukocidin (PVL) has been associated with invasive *Staphylococcus aureus* soft tissue and pneumonic infections.

**Methods:**

From September 2007 to January 2009 at Royal Perth Hospital we tested for the PVL gene in *S. aureus* isolates from an invasive site, a suspected PVL-related soft tissue infection and all MRSA isolates. We could access medical records for 141 PVL positive (PVL + ve) infections and compared these to a control group comprised of 148 PVL negative (PVL-ve) infections.

**Results:**

In the PVL + ve group 62 isolates were MRSA (48 were ST93-MRSA-IV) and 79 isolates were methicillin-sensitive *S. aureus,* and in the PVL-ve group 56 were MRSA (50 were WA-MRSA strains) and 92 were methicillin-sensitive *S. aureus*. We found the presence of PVL to be significantly associated with younger age, aboriginality, intravenous drug use, community acquisition, shorter length of hospital stay and lower mortality at 1 year. Overall PVL + ve infections more often required surgical intervention (73.0% versus 44.6%, p < 0.001) and were less often polymicrobial (8.5% versus 41.2%, p < 0.001). PVL + ve isolates were more often susceptible to clindamycin (87.9% versus 73.0%, p = 0.002).

**Conclusions:**

This study demonstrates that PVL + ve infections are associated with a distinct clinical picture, predominantly pyogenic skin and soft tissue infections often requiring surgery, disproportionately affecting patients who are younger, indigenous or with fewer health-care risk factors.

## Background

*Staphylococcus aureus* is a common human pathogen which causes a wide variety of infections. Over the past decades, *S. aureus* epidemiology has dramatically changed with the appearance of methicillin resistant strains in the community (CA-MRSA), when such strains had previously only been described in hospitals. Since its initial description [1], CA-MRSA has emerged throughout the world accounting for nearly 70% of *S. aureus* recovered from patients with skin and soft tissue infections attending an emergency department in the United States [2]. Although diverse clones are responsible for the emergence of CA-MRSA world-wide, the major global clones harbour the Panton-Valentine leukocidin (PVL) [3], a pore-forming toxin that disrupts host cell membranes and which is associated with recurrent boils [4], necrotising fasciitis [5] and necrotising pneumonia [6]; though is infrequently associated with asymptomatic carriage [7]. Naturally PVL has been well studied in CA-MRSA though our study contributes also to the expanding data on PVL in methicillin sensitive *S. aureus* (MSSA), and describes aspects of local Western Australian *S. aureus* epidemiology, clinical outcomes, antimicrobial susceptibility and distribution of MRSA types related to PVL.

## Methods

### Study design

Royal Perth Hospital is an adult tertiary centre with a 955-bed capacity. The Australian Collaborating Centre for *Enterococcus* and *Staphylococcus* Species (*ACCESS*) Typing and Research performs PVL testing on (a) all *S. aureus* grown from sites of invasive disease (e.g. tissue, bone/joint, blood), (b) *S. aureus* from some skin/soft tissue specimens (where there is some suggestion of aggressive infection such as abscess formation, determined by the microbiology scientist’s interrogation of the clinical notes and specimen description on the laboratory request form), (c) all MRSA isolates including nasal and skin colonisation swabs. For this study we excluded *S. aureus* colonisation, defined as *S. aureus* growth without need for surgery or antimicrobial therapy, and duplicate isolates from the same patient were not included. We collated data from all 170 PVL + ve isolates between September 2007 and January 2009. Medical records were unavailable for 7 cases and 22 (13.5%) colonising isolates were excluded, leaving 141 PVL + ve infections during the 17 month period. Of 1,146 PVL-ve isolates during the time period, 836 were MRSA cultured from nasal or skin colonisation swabs (72.9%). From the remaining 310 cases to approximate numbers in the PVL + ve group, 148 PVL-ve *S. aureus* infections were selected as “controls” by selecting every second isolate chronologically.

Demographics and co-morbidity data were collected from the medical record, and all cause mortality at 30-days and 1-year was determined from clinical information systems data-linked to the Western Australian Registry of Deaths. We recorded whether empirical antibiotic choice was effective (defined if the isolate was susceptible *in vitro* to any of the antibiotics prescribed), empirical use of protein synthesis inhibitors (lincosamide/linezolid), and lincosamide/linezolid use within 3 days of isolate acquisition. Infection was defined as “hospital acquired” if the isolate was collected more than 48 hours after admission and symptoms were not present on admission. “Healthcare associated” infection occurred in the community in the presence of at least one of the following three risk factors: (a) in the past 12 months a history of surgery, hospitalisation, dialysis or residence in a long term care facility, (b) presence of an invasive device at the time of infection onset, (c) a history of MRSA infection or colonisation. “Community acquired” infection occurred in the community without any of the above risk factors [8]. The study was approved by the Royal Perth Hospital Ethics Committee. Patient informed consent was waived in accord with guidelines of the National Health and Medical Research Council of Australia for a clinical audit [9].

### Microbiological methods

*S. aureus* was identified using routine laboratory methods (catalase-positive, tube coagulase-positive Gram-positive cocci). Antibiotic susceptibilities were determined by disk diffusion using CLSI methodology and breakpoints [10]. Methicillin resistance was determined by demonstrating cefoxitin resistance. Genes encoding PVL were detected with the nuclease (*nuc*) and *mecA* genes by an in-house real time polymerase chain reaction (PCR) assay using the Light Cycler 2.0 (Roche Diagnostics) as previously described [11]. Briefly a single colony incubating for less than 48 hours was first treated with lysostaphin, proteinase K and tris buffer. Cell lysate was added to the in-house PCR master mix containing 1× LightCycler Master Hybridization Probes mixture (Roche Molecular Biochemicals), 5 mM MgCl2, 0.5 μM forward and reverse primers and two FRET (Fluorescence Resonance Energy Transfer) probes (0.2 μM) each for PVL, mec and nuc genes. Two microlitres of sample was added to 18 microlitres of mastermix. We performed 45 cycles of PCR with 3 channel detection of the gene products followed by melt curve analysis. Six controls were used for each PCR run: MRSA PVL negative, MSSA PVL positive, MR coagulase negative Staphylococcus, MS coagulase negative Staphylococcus, a mutant MRSA PVL negative strain (which demonstrates low efficiency *nuc* PCR), and a negative control. A positive result for *PVL*, *mec* or *nuc* required exponential increase in fluorescence within 40 PCR cycles and a characteristic melting temperature. All MRSA and no MSSA isolates were typed by antibiogram, Christensen’s urea broth, and previously described coagulase gene PCR-Restriction Fragment Length Polymorphisms assay and contour clamped Homogenous Electric Field Electrophoresis [12]. Multi Locus Sequence Typing and Staphylococcal Chromosomal Cassette *mec* typing by PCR were performed on some isolates according to prior description [13].

### Statistical methods

Data was examined in IBM SPSS Statistics version 19 (SPSS Inc., Chicago, IL, USA). Continuous variables were examined by the t-test or Mann–Whitney U-test as appropriate and categorical variables by the Chi-Squared or Fisher’s exact test as appropriate. Logistic regression was performed entering all variables non-sequentially. For all statistical analysis a p-value <0.05 was considered to be significant.

## Results

Patients with PVL + ve *S. aureus* infections compared to the control group were younger (median age 37 vs 58 years, p < 0.001), more often male (72.3% vs 62.8%, p = 0.085), more commonly indigenous (34.0% versus 12.2%, p < 0.001), more often intravenous drug users (16.3% vs 6.8% respectively, p = 0.011), but were less likely to have haematological malignancy (0.0% vs 4.1%, p = 0.016) or a solid tumour (1.4% vs 9.5%, p = 0.003). Long-term care facility residents were rarely infected with PVL + ve strains, being 1.4% of PVL + ve cases compared to 14.9% of controls (p < 0.001). The majority of PVL + ve infections were community acquired in contrast to the control group which were often healthcare associated or hospital acquired infections (Table 1).

**Table 1:** **Epidemiological associations with PVL status**

**Characteristic**	**PVL positive (n = 141)**	**PVL negative (n = 148)**	**p-value**
Age (median)	37	58	<0.001
Male	102 (72.3)	93 (62.8)	0.085
Indigenous	48 (34.0)	18 (12.2)	<0.001
Intravenous Drug Use	23 (16.3)	10 (6.8)	0.011
Haematological malignancy	0 (0)	6 (4.1)	0.016
Solid malignancy	2 (1.4)	14 (9.5)	0.003
Diabetes Mellitus	31 (22)	44 (29.7)	0.133
Immunosuppressive treatment	7 (5)	14 (9.5)	0.141
HIV positive	3 (2.1)	2 (1.4)	0.613
Pre-existing skin condition	4 (2.8)	4 (2.7)	0.945
Hospitalisation in the past year	50 (35.5)	92 (61.2)	<0.001
Long term care facility resident	2 (1.4)	22 (14.9)	<0.001
“Community acquired” infection	87 (61.7)	35 (23.6)	<0.001
“Healthcare associated” infection	46 (32.6)	78 (52.7)	<0.001
“Hospital acquired” infection	8 (5.7)	35 (23.6)	<0.001

Number (%).

Skin and soft tissue was the most common site of infection in both groups, being significantly more common in the PVL + ve compared to the PVL-ve group (81.6% vs 63.5%, p < 0.001), and abscesses accounted for a larger percentage of these infections in the PVL + ve group versus controls (87.0% compared to 41.5% respectively, p < 0.001). Bacteraemia occurred in 50 cases, of which 13 (26.0%) were PVL + ve (p < 0.001). Bone and joint infections were the third most common site of infection being less common in the PVL + ve than the control group (3.5% vs 8.8% respectively, p = 0.066). Seven (5.0%) lung infections were due to PVL + ve *S. aureus* (4 empyema, 1 lung abscess, 2 pneumonia) compared to 3 (2.0%) due to PVL-ve *S. aureus* (2 pneumonia and one empyema, p = 0.172). For 12 (8.5%) PVL + ve infections another pathogen was grown from the same specimen compared to 61 (41.2%) of the control group. This difference was most marked for skin and soft tissue infections though a trend was also seen in bloodstream and bone/joint samples (Table 2).

**Table 2:** **Specimen type by PVL status**

**Specimen type**	**Subcategory**	**PVL positive (n = 141)**	**PVL negative (n = 148)**	**p-value**
All	Polymicrobial	12 (8.5)	61 (41.2)	<0.001
Skin/soft tissue	Total	115 (81.6)	94 (63.5)	<0.001
	Polymicrobial	10 (8.7)	51 (54.3)	<0.001
*(a) Cellulitis/wound swab*	Total	15 (13.0)	55 (58.3)	<0.001
	Polymicrobial	5 (33.3)	33 (60.0)	0.006
*(b) Abscess soft tissue*	Total	100 (87.0)	39 (41.5)	<0.001
	Polymicrobial	5 (5.0)	18 (46.2)	<0.001
Blood	Total	13 (9.2)	37 (25.0)	<0.001
	Polymicrobial	0 (0.0)	3 (8.1)	0.290
Bone/Joint	Total	5 (3.5)	13 (8.8)	0.066
	Polymicrobial	0 (0.0)	5 (38.5)	0.103
Lung	Total	7 (5.0)	3 (2.0)	0.172
Renal	Total	0 (0.0)	1 (0.7)	0.328

Number (%).

Antimicrobial susceptibility results are shown in Table 3. Of note, 87.9% of PVL + ve isolates tested susceptible (includes a negative D-test) to clindamycin compared with 71.6% of the control isolates (p = 0.002). Erythromycin and mupirocin susceptibility rates were also higher for PVL + ve strains compared to controls. Overall, only 80.1% of MRSA isolates were susceptible to clindamycin (data not shown).

**Table 3:** **Antibiotic susceptibility by PVL status**

**Antibiotic**	**PVL positive (n = 141)**	**PVL negative (n = 148)**	**p-value**
Methicillin	79 (56.0)	92 (62.2)	0.289
Clindamycin	124 (87.9)	106 (71.6)	0.002
Erythromycin	122 (86.5)	105 (70.9)	0.005
Mupirocin	139 (98.6)	137 (92.6)	0.040

Number (%).

Queensland clone (ST 93-IV, n = 48) was the predominant PVL + ve MRSA strain, with smaller numbers of Western Samoan Phage Pattern strains (ST30-IV, n = 5), USA 300 (ST8- IV, n = 4), WA1-MRSA strains (ST1-IV, n = 3), Taiwan strain (ST59-VT, n = 1) and EMRSA-15 (ST22-IV, n = 1). For PVL-ve MRSA isolates, most were WA-MRSA strains (WA-MRSA-1 [ST1- IV], WA-MRSA-2 [ST78-IV], WA-MRSA-3 [ST5-IV], WA-MRSA-75 [ST45-IV], n = 50) with few overseas strains: EMRSA-15 (ST22-IV, n = 6). No MSSA isolates were typed.

Empirical antimicrobial therapy was effective in 75 (53.2%) PVL + ve cases and 90 (60.8%) control cases (p = 0.191). Cases not receiving effective empiric therapy were predominantly because of unexpected methicillin resistance (103 cases, 83.1%, OR 9.1 [4.7-10.7 95% CI]) or as infections were polymicrobial (39 cases, 31.5%, OR 1.3 [0.99-1.70 95% CI]). Lincosamides or linezolid (protein synthesis inhibitors) were more frequently added to therapy and surgery was more often required (73.0% versus 44.6%, p < 0.001) for PVL + ve infections compared to controls (Table 4).

**Table 4:** **Outcomes by PVL status**

**Management or Outcome**	**PVL positive (n = 141)**	**PVL negative (n = 148)**	**p-value**
Initial antibiotics effective	75 (53.2)	90 (60.8)	0.191
Lincosamide/linezolid initial Tx^a^	9 (6.4)	4 (2.8)	0.141
Lincosamide/linezolid 3d^b^	29 (20.8)	10 (6.8)	<0.001
Surgery performed	103 (73.0)	66 (44.6)	<0.001
HDA/ICU admission^c^	8 (5.7)	17 (11.5)	0.079
Median length of stay-days (range)	5.0 (0–81)	13.5 (0–638)	<0.001
Death within 30 days	4 (2.8)	10 (6.8)	0.121
Death within 1 year	9 (6.4)	29 (19.6)	0.001

^a^Lincosamide or linezolid prescribed in the initial therapy.

^b^Lincosamide or linezolid added to therapy within 3 days of isolate collection.

^c^High dependency area or intensive care unit admission.

Number (%).

Rates of admission to high dependency unit and/or intensive care unit were lower (5.7% vs 11.5%, p = 0.079) and length of hospital stay was significantly shorter (median 5.0 vs 13.5 days, p < 0.001) for PVL + ve infections compared to controls. Both 30 day and 1 year all cause mortality were lower with PVL + ve infections than for PVL-ve infections (2.8% vs 6.8%, p = 0.121; 6.4% vs 19.6%, p = 0.001) (Table 4). The 30-day mortality was not influenced by adjunctive protein synthesis inhibitor therapy (2.7% vs 5.3% for PVL + ve infections and controls, respectively; p = 0.534). Interestingly, outcome was not influenced by initiation of effective empiric therapy (data not shown).

We performed a logistic regression analysis of PVL status incorporating variables aboriginality, age, intravenous drug use (IVDU), community acquisition, abscess, surgery and hospital length of stay. Statistically significant independent associations with PVL were found for aboriginality (OR 3.89, 1.83-8.25 95% CI, p < 0.001), age (OR 0.96, 0.94-0.98 95% CI, p < 0.001), community acquisition (OR 2.47, 1.32-4.62 95% CI, p = 0.005), abscess (OR 3.45, 1.78-6.69 95% CI, p < 0.001), but were not found for IVDU (OR 1.67, 0.64-4.37 95% CI, p = 0.29), surgery (OR 1.77, 0.92-3.41 95% CI, p = 0.089), or hospital length of stay (OR 0.98, 0.97-1.00 95% CI, p = 0.100).

For the subset of MRSA isolates, PVL was significantly associated with younger age (median 37 vs 50 years, p < 0.001), IVDU (p = 0.047), abscess formation (p < 0.001), community acquisition (p < 0.001), monomicrobial infection (p < 0.001), surgery (p = 0.001), shorter length of stay (median 3 vs 10 days, p < 0.001), but not significantly associated with aboriginality (p = 0.091).

For the subset of invasive isolates, PVL was significantly associated with aboriginality (p = 0.001), but not significantly associated with age (median 41 vs 45 years, p = 0.378), community acquisition (p = 0.171), IVDU (p = 1.000), surgery (p = 0.433), or length of stay (median 5 vs 7 days, p = 0.300).

## Discussion

We found PVL + ve *S. aureus* less frequently associated with colonisation than controls (13.5% vs at least 72.9%, respectively). A Polish group has replicated these findings, showing that 85.0% of *S. aureus* isolates causing furunculosis were PVL + ve, compared to asymptomatic blood donors where less than 1.0% of nasal swabs yielded positive strains [14]. Likewise, the prevalence of PVL + ve *S. aureus* colonisation was 4.5% in indigenous and rural Malaysian communities [15]. A north-east Australian study of nasal colonisation of volunteers and patients seeing their general practitioner with a non-infectious illness (total sample size 699) showed that only 1.0% (2/202) of *S. aureus* isolates recovered were PVL + ve, in contrast to results showing 55.0% of non-multiresistant MRSA and 13.0% of MSSA clinical isolates in this region were PVL + ve [16]. Conversely, a New Zealand study of clinical isolates versus asymptomatic nasal isolates of methicillin sensitive *S. aureus* found the prevalence of PVL + ve strains was no different between these groups (37.0% vs 31.0%, p = 0.33) [17]. Heterogeneity between such studies may be due to differing ability of various PVL + ve clones to cause colonisation.

Our study shows strong associations between PVL + ve *S. aureus* infections and younger age, male gender, aboriginality, intravenous drug use and community acquired infection compared to healthcare-associated infection. Conversely for PVL-ve infections, healthcare-associated epidemiological factors such as malignancy or nursing home residency were significantly more common. These associations were true for MRSA and MSSA. Our findings support other recent reports that include MSSA: A study from the Northern Territory of Australia of 223 PVL + ve and 255 PVL-ve *S. aureus* isolates (methicillin susceptible and resistant) showed that presence of PVL was associated with younger age, fewer co-morbidities and community onset [18]. Likewise, the aforementioned Auckland study of 124 PVL + ve and 211 PVL-ve MSSA isolates found PVL associated with younger age, Pacific ethnicity, surgical drainage needed and community onset [17].

One hypothesis for the higher rates of PVL + ve *S. aureus* infections amongst younger patients is that older patients through previous exposure might have developed immunity to this toxin. Indeed, in the 1950s, a phage 80/81 strain, now MLST ST30, became pandemic causing outbreaks of skin and soft tissue infections and pneumonia, especially in children [19]. A leukotoxin was thought responsible for the virulence and transmissibility of the strain. One postulate suggests this epidemic had waned by the mid 1960s due to the advent of penicillinase-resistant penicillins, but potentially the epidemic waned from rising immunity to PVL within the general population; supporting this concept it was shown the toxic effect of PVL on cells could be neutralised by immunoglobulins from patients convalescing from infections caused by this strain [20]. Furthermore, Gauduchon et al. have reported increasing prevalence of antibodies to PVL with increased age, further supporting an immunity hypothesis [21].

Our study shows that PVL + ve *S. aureus* is strongly associated with skin and soft tissue infection, predominantly abscesses. On the contrary, there is a suggestion that PVL + ve *S. aureus* infrequently caused bacteraemia or bone and joint infection. These findings have consistently been reported in the literature [2,4,18,22,23] summarised in the meta-analysis by Shallcross et al. [24], and together with PVL + ve *S. aureus* strains tending to affect younger patients with less co-morbidities, partly explains the overall lower mortality from PVL + ve *S. aureus* disease [17]. However, PVL + ve *S. aureus* strains more often lead to relapse of infection [14] and can cause epidemics of skin/soft tissue infections as reported by rising Emergency Department attendances for furunculosis, creating significant disease burden for healthcare systems [25]. Whilst PVL + ve *S. aureus* strains generally cause a relatively benign disease spectrum, life threatening infections such as necrotising pneumonia are well documented [6,26]. This particular manifestation of PVL + ve *S. aureus* disease is rare, presumably because additional co-factors are needed to mediate this severe clinical picture, for example preceding viral infection [6]. Unfortunately, our sample size was too small to substantiate any difference between PVL + ve and PVL-ve *S. aureus* lung infections.

We believe our study failed to demonstrate any benefit of adding a protein synthesis inhibitor (e.g. lincosamide or linezolid) to therapy of PVL + ve *S. aureus* infections because surgical drainage of abscesses, the commonest presentation of these infections, is the mainstay of their treatment with antibiotics playing only a limited role in treatment outcomes. Nonetheless, addition of a protein synthesis inhibitor has been recommended for severe invasive PVL + ve *S.aureus* lung disease [27].

The most significant limitation of this study is the potential for selection bias. Crucially we have selected skin/soft tissue *S. aureus* isolates for PVL testing because we expected to find the PVL gene with a history of abscess formation, based on previous literature. This finding is borne out in the association of PVL and abscess formation in the study, however there is a risk we have biased the PVL + ve group with abscess specimens falsely elevating the percentage of skin/soft tissue to invasive infections, potentially confounding bacteraemia rates or outcome data as examples. A multivariate analysis including abscess as a variable and subset analysis of isolates sent for PVL testing for different reasons (MRSA or invasive isolates) are attempts to account for this selection bias, and many variables associated with PVL in the primary analysis continued to show significant associations in these analyses. Ultimately testing all *S. aureus* laboratory isolates for the PVL gene would have been more ideal. Due to the dominance of ST 93-IV in the PVL + ve MRSA group, it is possible other factors associated with this clone influenced outcomes in this group. Additionally, lack of MSSA typing leads to an incomplete understanding of complete clonal makeup of either group. Other limitations of the study include: the study was performed in an adult teaching hospital in Perth, Western Australia and might not necessarily represent the clinical and molecular epidemiology of invasive *S. aureus* infection in other jurisdictions in Australia or in the paediatric or Aboriginal populations; the study was non-interventional and may lack statistical power to detect differences for some outcomes; clinical data relied on hospital medical records rather than a specific case report form and therefore we could have underestimated the frequency of risk factors and additional data points.

## Conclusions

In summary, our study demonstrates that PVL + ve MRSA and MSSA are associated with a specific clinical picture, predominantly pyogenic skin and soft tissue infections which are often monomicrobial and require surgery, more often involving patients who are younger, indigenous or with fewer healthcare risk factors. Whether immunisation or new therapeutic strategies can overcome this worldwide epidemic remains to be seen.

## Abbreviations

CA-MRSA: Community acquired methicillin resistant *Staphylococcus aureus*
CLSI: Clinical Laboratory Standards Institute
IVDU: Intravenous drug use
MRSA: Methicillin resistant *Staphylococcus aureus*
MSSA: Methicillin sensitive *Staphylococcus aureus*
PCR: Polymerase chain reaction
PVL: Panton Valentine Leucocidin
ST: Sequence type
WA: Western Australia
